# Social Support, Isolation, Loneliness, and Health Among Older Adults in the PRISM Randomized Controlled Trial

**DOI:** 10.3389/fpsyg.2021.728658

**Published:** 2021-10-05

**Authors:** Sara J. Czaja, Jerad H. Moxley, Wendy A. Rogers

**Affiliations:** ^1^Division of Geriatrics and Palliative Medicine, Center on Aging and Behavioral Research, Weill Cornell Medicine, New York, NY, United States; ^2^College of Applied Health Sciences, University of Illinois Urbana-Champaign, Champaign, IL, United States

**Keywords:** social Isolation, loneliness, older adults, technology, health

## Abstract

**Objectives:** Social isolation and loneliness are serious public health issues given the association with negative physical, mental; and cognitive health outcomes and increased risk for mortality. Due to changes in life circumstances many aging adults are socially isolated and experience loneliness. We examined the relationships among four correlated but distinct constructs: social network size, social support, social isolation, and loneliness as they relate to indices of health and wellbeing among diverse subpopulations of older adults. Guided by WHO’s International Classification of Functioning, Disability and Health (ICF) we also examined factors that predict loneliness and social isolation.

**Methods:** Analyses of baseline data from sample of older adults who participated in an intervention trial that examined the beneficial effects of a software system designed to support access to resources and information, and social connectivity. Participants included 300 individuals aged 65–98, who lived alone, were primarily of lower socio-economic status and ethnically diverse. Participants completed a demographics questionnaire, self-report measures of health, depression, social network size, social support, and loneliness.

**Results:** Loneliness was strongly associated with depression and self-ratings of health. In turn, greater social isolation and less social support were associated with greater loneliness. Social isolation was associated with depression and lower self-ratings of health. The association between social isolation and health was mediated by loneliness. Individuals in the older cohorts (80+) reported less social support. With respect to loneliness, having a smaller social network, more functional limitations, and limitations in engaging meaningful activities was associated with higher levels of loneliness and greater social isolation.

**Conclusion:** The findings underscore the importance of social connectively to wellbeing for older adults and suggest that those in the older cohorts, who have a small social network, and with greater physical and functional impairments may be particularly vulnerable to being socially isolated and lonely. The findings provide guidance for future interventions. In this regard, we discuss how Information and Communication Technologies (ICTs) may be used to promote social connectivity and engagement. Strategies to make the usability and availability of these applications for aging adults are highlighted.

## Introduction

The number of people in the United States aged 65+ will increase to about 98 million by 2060, with the fast-growing cohort of the “oldest old” (85+) projected to number 14.6 million by 2040 (Administration for Community Living, 2020). The burgeoning population of older adults especially those in the “oldest old” cohort (85+) has given rise to concern about the need for strategies to maintain the health and independence of this population.

Recently, increased attention has been directed toward social isolation and loneliness as significant health risks for aging adults. Changes in life circumstances, such as retirement, loss of partners or friends, financial circumstances, health declines, and mobility challenges make older people vulnerable to becoming isolated and lonely. Current estimates suggest that approximately, one-quarter of community dwelling adults aged 65 and older are socially isolated (Anderson and Thayer, 2018) and that almost half (43 percent) of those 60 and older reported feeling lonely (Cudjoe et al., 2020). The growing concerns about social isolation and loneliness among aging adults is underscored by the recent consensus study by National Academies of Science Engineering and Medicine (2020) that focused on social isolation and loneliness in older adults. One conclusion was that social isolation and loneliness play as large a role as other well-established risk factors for negative health consequences such as obesity and smoking (Donovan and Blazer, 2020).

Substantive results in the literature link social isolation and loneliness to heightened risk for physical difficulties, mental health problems, cognitive deficits, functional declines, and mortality (e.g., Cacioppo et al., 2010; Holt-Lunstad et al., 2010; Aylaz et al., 2012; Perissinotto et al., 2012; Valtorta et al., 2016; Hakulinen et al., 2018; Jeuring et al., 2018; Domenech-Abella et al., 2019; Read et al., 2020). Data from the English Longitudinal Study of Aging indicated that loneliness is a significant, independent predictor of dementia (Rafnsson et al., 2017).

Current models of “successful aging” (e.g., Rowe and Kahn, 1988; Kahana and Kahana, 1996, 2001) posit that engagement in productive and social activities is key to successful aging. Social engagement is multifaceted and includes personal relationships, connections with the community (e.g., neighborhood), and engagement with society. Personal relationships provide social support and opportunities for reciprocal communication and feeling valued or mattering. Connection with the community fosters a sense of belonging; participation in society provides opportunities to contribute and engage with ideas. Recently, the term social capital has been used in discussions of social engagement and generally refers to resources available to individuals and groups through social connections and their community (Cannuscio et al., 2003).

With the increasing number of adults in the older cohorts and other demographic trends, such as geographical dispersion of families and changes in family structures, social isolation will continue to be an issue in the foreseeable future. This is especially true in light of the COVID-19 pandemic where stay-at-home requirements curtailed opportunities for face-to-face interactions, participation in social activities, and access to social networks and support. Much is being written about the potential implications of the enforced social restrictions on mental health and well-being (e.g., Armitage and Nellums, 2020; Wu, 2020). As noted by the NASEM report (2020) social isolation and loneliness are modifiable risk factors for health and although much has been written about the link between social isolation and loneliness and health consequences, the literature on effective interventions to remediate existing problems with social isolation and loneliness and prevent further incidence for vulnerable individuals is limited. Development of efficacious intervention strategies requires understanding how to best assess social isolation and loneliness; the prevalence and predictors of isolation and loneliness; and variations within subpopulations.

Social isolation and loneliness are distinct constructs, which are related but only moderately correlated. Social isolation can be measured objectively and refers to social network size and the existence and interconnections among different social ties. Loneliness is subjective and refers to a person’s self-perceived lack of social support and companionship. Social support refers to the provision of emotional, instrumental, or informational resources to help an individual cope with stress and life events (Cohen, 2004) and is related to social connectivity. However, the provision of support does not necessarily imply that an individual is satisfied with the support received. There are various measures of these constructs available, which contributes to the inconsistencies among findings regarding the prevalence of social isolation, loneliness, and social support among older people and association of these variables with health and well-being outcomes. Additionally, few studies have examined these factors conjointly. Coyle and Dugan (2012) stressed that it is important to distinguish between social isolation and loneliness when examining health outcomes in older adults as they are different constructs and may have differential impacts on indices of health.

In this study, we had the unique opportunity to examine the relationships among aspects of social engagement and the relationships of these factors to health outcomes among a large and diverse sample of older adults who live alone in the community. Although living alone has been associated with higher rates of isolation and loneliness, the relationship between living alone and these factors is complex. As noted by Perissinotto and Covinsky (2014), we cannot assume that people who are living alone are lonely or lacking in social connectivity and support. We examined how these relationships vary among cohorts of older adults (younger-old and older-old) as there is heterogeneity across older age cohorts on numerous variables. For example, those in older cohorts are more likely to have fewer social connections and greater role limitations due to changes in life circumstances, health, and mobility issues. In addition, we examined how social isolation and loneliness influence physical and emotional health outcomes and cognition. We examined these outcomes separately as the literature suggests that the predictors of these outcomes may vary. Understanding the unique factors associated with distinct outcomes is important to the design of intervention strategies. Finally, guided by the WHO Model of Functioning, Disability, and Health (World Health Organization, 2002), we examined personal (e.g., income, age), community (e.g., social network), and health factors (e.g., health conditions) that are associated with social isolation and loneliness. The WHO model provides a framework for understanding health outcomes and determinants. Based on the substantive literature, examining the impacts of social isolation and loneliness, we hypothesized that loneliness and social isolation would be independent predictors of depressive symptoms, health, and cognition. We also wished to examine if social support and loneliness impacted our outcomes through different mechanisms. In addition, we hypothesized, given that an important aspect of loneliness is a sense of not being integrated into a social environment (Tiikkainen and Heikkinen, 2005), that social support would be related to loneliness such that those with lower perceived social support would report higher levels of loneliness. Further, as we had measures of both the structural and functional aspects of social connectivity, we hypothesized that social network size and social isolation would be related to perceived social support.

## Materials and Methods

The sample for the analyses was comprised of participants in the Personal Reminder, Information, and Social Management (PRISM) randomized field trial (Czaja et al., 2015, 2018), which examined the benefits of a computer system designed to support access to social connectivity and support access to information, and engagement among older people. We present a summary of the PRISM trial as the methods and the main outcomes of the trial have been previously reported (Czaja et al., 2015, 2018).

### Protocol

Potential participants contacted the site study coordinator and completed a telephone screening that assessed eligibility. For those eligible, a home baseline assessment was scheduled. During this assessment participants provided informed consent. An assessor trained and certified in the study protocol administered the assessment. Participants were compensated $25 for the assessment. Participants were then randomly assigned to the PRISM condition or a Binder Control condition. Those in the PRISM condition received hardware and software training and had the PRISM system installed their home for 12 months. Those in the Binder Control condition received a binder containing content that paralleled the PRISM system in a non-electronic form (e.g., paper resource guides, paper calendar). PRISM included Internet access (with vetted links to sites such as NIH SeniorHealth.Gov); an annotated resource guide; a dynamic classroom feature; a calendar; a photo feature; email; games; and online help. Participants also completed 6- and 12-month follow-up assessments administered by assessors blinded to treatment condition. The Institutional Review Boards at the sites approved the study and all participants provided informed written consent. Here we report on data from the baseline assessment from all study participants.

### Sample

We recruited 300 older adults at risk for social isolation, operationalized as: lived alone, did not spend more than 10 h each week at a Senior Center, did not work or volunteer for more than 5 h per week, and had minimal computer and Internet experience in the past 3 months. Eligible participants were 65 years of age or older, spoke English, and could read at the 6th grade level. Participants were recruited through advertisement and various outreach methods [e.g., churches, community organizations from the Atlanta (GA), Miami (FL), and Tallahassee (FL) regions of the United States]. The sample was primarily female (78%) and ranged in age from 65 to 98 years (*M* = 76.15, *SD* = 7.4); 33% of the sample was ≥80 years and 15% were 85+ years. Participants were ethnically diverse (46% non-White), 89% had annual household incomes <$30,000, and 39% had high school or less education (Czaja et al., 2015).

### Measures

The full list of measures collected in the trial is available in Czaja et al. (2015). The present study focused on the indices of: social isolation (Friendship Scale – Hawthorne, 2006; α = 0.75); loneliness (The UCLA Loneliness Scale – Russell, 1996; α = 0.91); social support (Interpersonal Support Evaluation List, ISEL; Cohen et al., 1985); Social Network Size (Lubben Social Network Index; Lubben, 1988; α = 0.85); depressive symptoms (20 item Center for Epidemiological Depression Scale, CESD; Radloff, 1977; Irwin et al., 1999; α = 0.87); and a self-rating of overall health. We used the common single item self-rating of health (“In general, would you say your health is” with the response items “excellent, very good, good, fair, or poor”), which has predictive validity with respect to objective measures of health status such as disease prevalence (i.e., Wu et al., 2013). Life Space was measured by the Life Space Questionnaire [Stalvey et al., 1999; κ = 0.80 (as cited by Stalvey et al., 1999)], wherein participants answer nine questions related to their mobility during the past 3 days (e.g., travel, getting out and about on a daily basis to places such as immediate neighborhood or town). Each item is rated “yes/no” and a score is computed by summing across the items. A higher score indicates greater mobility. Life Engagement was measured by the Life Engagement Test (Scheier et al., 2006; α = 0.77), a six-item scale, which measures that extent to which a person engages in activities that are personally valued. A lower score indicates higher engagement. We created a variable to indicate functional disabilities by summing responses to a question regarding activity limitations (e.g., bathing, stair climbing, walking, engaging in sports activities) due to health (range 0–10). We created a variable to indicate health conditions by summing responses to a question regarding the presence of a health condition (e.g., diabetes, arthritis, hypertension) (range = 0–11). Cognition was measured by a latent construct (see section “Results”) comprised of a measure of processing speed [Digits Symbol Substitution, Weschler, 1981; α = 0.96 (as reported in Lövdén et al., 2005)], reasoning [Letter Sets, Ekstrom et al., 1976; α = 0.77 (as reported in Ekstrom et al., 1976)], and attention/executive function [Trail Making Test A and B, Reitan, 1958; α = 0.84 (as reported in Dikmen et al., 1999)].

## Analysis

For the structural equation models, we used Mplus, which by default handles missing data using full information maximum likelihood and uses all available raw data to estimate missing data for a given case. This approach does well at retrieving correct parameters in simulation (Enders and Bandalos, 2001). Social isolation was assessed using the Friendship Scale and higher scores mean less socially isolated. Model fit statistics are reported using X^2^, root mean square error of approximation (RMSEA), comparative fit index (CFI), Tucker-Lewis index (TLI), and the standardized root mean residual (SRMR; Kline, 2011).

Each structural equation model included one latent variable for social support, computed by combining the three subscales of the ISEL scale. The model for cognition included a latent variable formed from the Digit Symbol Substitution Test, the Letter Sets Test, and the Trails A test. Trails B was considered for model inclusion but was excluded because it was highly collinear with Trails A. Thus, including both Trails A and Trails B would damage model fit.

We used a path model to examine the extent to which loneliness mediated the relationship between social support, social network size, and social isolation with the dependent variables of depressive symptoms, self-rated health, and latent cognition. We examined whether social support mediated the relationship between social isolation and social network size and depression, health, and cognition. Indirect effects are reported as well as the total indirect effect, which examines if the sum of the indirect paths is statistically significant, and specific indirect effects, where each individual path, is analyzed separately. Significant specific paths are informative even in the absence of a total effect (Rucker et al., 2011).

We then conducted a multiple group analysis of the structural equation model, which involved testing the efficacy of adding increasing levels of equality constraints on the parameters for the younger-older adults and the older-old adults. We started with a model where the groups were allowed to differ on most parameters. Equality constraints were then added to various families of parameters. This allowed us to sequentially test if the means of the variables, the factors loadings, the paths, and the residual errors of the groups differed. The change in X^2^ was used to assess model fit. If the change in X^2^ was statistically significant when a constraint forcing the two groups to be equal was added, this would suggest the two groups differed on a parameter (e.g., the mean of social support) or on a set of parameters.

## Results

Table 1 provides the inter-correlations and descriptive statistics for the variables included in the present analyses. The group means for the older-old (defined as 80+ years) (*n* = 101) and the younger-old (*n* = 199) samples as well as univariate contrasts are provided in Table 2. We had incomplete data for some of the variables: loneliness (*n* = 299), health (*n* = 298), social isolation (*n* = 299), and social network size (*n* = 299).

**TABLE 1:** Intercorrelations and descriptive statistics of study variables.

Variable	Mean	1	2	3	4	5	6	7	8	9	10	11	12
1. ISEL appraisal	8.71	2.65											
2. ISEL tangible	8.74	0.56**	2.77										
3. ISEL belonging	7.66	0.54**	0.60**	2.75									
4. Age	76.15	−0.10	−0.05	−0.16**	7.37								
5. Social isolation	19.24	0.50**	0.47**	0.46**	0.07	3.93							
6. Social network size	26.22	0.39**	0.42**	0.39**	−0.06	0.37**	7.39						
7. UCLA loneliness	39.51	−0.54**	−0.57**	−0.62**	−0.01	−0.72**	−0.44**	10.00					
8. Health	3.03	0.16**	0.18**	0.22**	0.02	0.29**	0.18**	−0.35**	0.86				
9. CES-D	11.11	−0.34**	−0.31	−0.33**	−0.08	−0.65**	−0.22**	0.57**	−0.30**	9.03			
10. Digit symbols	34.95	0.08	0.01	0.08	−0.14*	0.07	0.06	−0.06	0.05	−0.002	11.31		
11. Letter sets	8.59	0.04	0.05	0.04	−0.16**	0.07	0.11	−0.09	0.01	−0.08	0.45**	5.23	
12. Trails A	4.02	−0.06	−0.08	−0.11	−0.14*	−0.03	−0.08	0.03	−0.11	−0.02	−0.60**	−0.34**	0.37

*Standard deviations are displayed on the diagonal (N = 300).*

*ISEL, Interpersonal Support Evaluation List; CES-D, Center for Epidemiologic Studies Depression Scale.*

*p < 0.05, **p < 0.01.

**TABLE 2:** Group means and standard deviations for the younger old (65–79) and the older old (80–98) participants (*N* = 300).

	Younger old		Older old		
Variable	Mean	STD	Mean	STD	*t*-test
Age	71.85	4.27	84.62	4.13	24.73**
Health	3.05	0.85	3.00	0.90	−0.47
Depression	11.36	8.86	10.62	9.40	−0.67
ISEL appraisal	8.89	2.56	8.35	2.80	−1.68
ISEL tangible	8.83	2.67	8.55	2.97	−0.83
ISEL belonging	7.94	2.73	7.10	2.70	−2.54*
Social isolation	19.06	4.07	19.59	3.64	1.19
Social network score	26.75	7.05	25.19	7.96	−1.80
UCLA loneliness	39.45	10.01	39.65	10.02	0.07
Digit symbol	35.01	10.73	34.85	12.45	−0.11
Letter sets	8.97	5.12	7.82	5.40	−1.72
Trails A	5.00	0.51	5.08	0.46	0.86

*ISEL, Interpersonal Support Evaluation List; CES-D, Center for Epidemiologic Studies Depression Scale.*

*p < 0.05, **p < 0.01.

### Structural Equation Model of Depressive Symptoms

**FIGURE 1:**
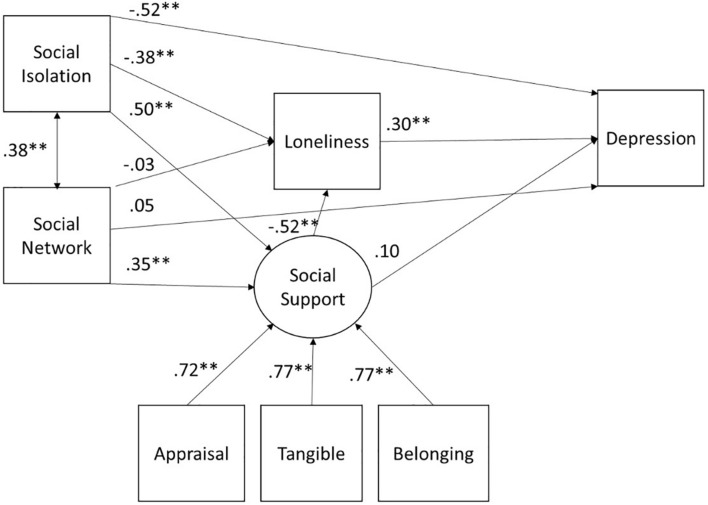
SEM for depression for the full sample. All paths are denoted with the standardized beta. ^∗^*p* < 0.05, ^∗∗^*p* < 0.01.

As shown in the Table 3, model fits were generally excellent: X^2^(8) = 11.00, *p* = 0.20, RMSEA = 0.04 90% CI (0.00, 0.08), CFI = 1.00, TLI = 0.99, and SRMR = 0.02 for the model without age analyzed as a grouping variable. Figure 1 shows the complete model for depressive symptoms without the age groupings. As shown, those with smaller social networks reported being more socially isolated and having less perceived social support. In turn, greater social isolation and less social support were related to higher degrees of loneliness. Importantly, higher levels of loneliness and greater social isolation independently predicted higher levels of depressive symptoms.

**TABLE 3:** Results of structural equation models for depression.

Model	df	X^2^	SRMR	RMSEA	TLI	CFI	Δdf	ΔX^2^
**No age model**	8	11.00	0.015	0.035	0.99	0.997		
**Multi-group factor analysis**								
Model 1. Paths, means (LV, IVs, DV), variances free	20	33.85*	0.044	0.068	0.97	0.99	–	–
Model 1 vs. Model 2	–	–	–	–	–	–	1	7.87**
Model 1 vs. Model 3	–	–	–	–	–	–	3	6.96
Model 2. Paths, means (IVs, DV), variances free	21	41.72**	0.084	0.081	0.96	0.98	–	–
Model 2 vs. Model 4	–	–	–	–	–	–	3	4.34
Model 3. Paths, means (DV, LV), variances free	23	40.81*	0.049	0.072	0.97	0.98	–	–
Model 3 vs. Model 4	–	–	–	–	–	–	1	5.25*
Model 3 vs. Model 5	–	–	–	–	–	–	1	8.29**
Model 3 vs. Model 6	–	–	–	–	–	–	2	13.89**
Model 3 vs. Model 7	–	–	–	–	–	–	10	15.10
Model 4. Paths, means LV, variances free	24	46.06**	0.049	0.078	0.97	0.98	–	–
Model 5. Paths, means DV, variances free	24	49.10**	0.092	0.084	0.96	0.97	–	–
Model 6. Paths variances free	25	54.69**	0.091	0.089	0.95	0.97	–	–
Model 7. Variances, means (DV, LV) free	33	55.90**	0.096	0.068	0.98	0.94	–	–
Model 7 vs. Model 8	–	–	–	–	–	–	8	13.05
Model 8. Means (DV, LV) free	41	68.95**	0.10	0.067	0.97	0.97	–	–
Model 8 vs. Model 9	–	–	–	–	–	–	1	0.02
*Model 9. Means (LV) free*	*24*	*68.97*	*0.10*	*0.065*	*0.97*	*0.97*	*–*	*–*

*The first model is without age. The rest of the models are multiple group analyses testing measurement equivalence on different sets of parameters. The best fitting multiple group model as determined by ΔX^2^ is in italic.*

*SRMR, Standardized Root Mean Square Residual; RMSEA, Root Mean Square Error of Approximation; TLI, Tucker-Lewis Index; CFI, Comparative Fit Index; Δdf, change in df; ΔX^2^, change in X^2^; LV, latent variable; IV, Independent Variable; DV, Dependent Variable.*

*p < 0.05, **p < 0.01.

We tested the mediation effects of social network size and social isolation to depressive symptoms through social support and loneliness. There was a significant direct effect of social isolation (β = −0.14, *z* = −3.16, *p* = 0.002) on depressive symptoms, meaning greater social isolation predicted more depressive symptoms. The indirect effect of social isolation on depressive symptoms through loneliness was significant (β = −0.12, *z* = −3.52, *p* < 0.001). The path of social isolation to depressive symptoms through social support and then loneliness was also significant (β = −0.08, *z* = −2.99, *p* = 0.003).

The total indirect effect of social network size (β = −0.026, *z* = −0.80, *p* = 0.43) on depressive symptoms was not significant. However, the indirect effect of social network size on depressive symptoms mediated through social support and then loneliness was statistically significant (β = −0.05, *z* = −2.51, *p* = 0.01). Social support had a statistically significant indirect effect on depressive symptoms (β = −0.33, *z* = −3.10, *p* = 0.002) through loneliness.

Overall, social isolation and loneliness had significant direct effects on depressive symptoms. In addition, the effects of social isolation and social support on depression were mediated by loneliness. Further, those with smaller social networks perceived less social support, which was in turn related to greater loneliness.

### Multiple Groups Analysis of Depressive Symptoms

We replicated the previous structural equation model for the two sub-groups of older adults (65–79 and 80–98). Table 3 presents the full multiple group analyses that tests measurement equivalence between the two groups of older adults. If measurement equivalence is not established no latent comparison can be meaningfully made. The constraints tested were path coefficients. We tested the means of the dependent variable of depressive symptoms, the independent variables (social isolation, social network size, and loneliness), and the latent variable of social support, and compared the variances. For depressive symptoms, constraining the latent variable of social support to be equal and constraining depressive symptoms to be equal significantly decreased model fit suggesting the two groups varied significantly on those variables. As more constraints were added such as fixing all paths and variances to be equal, depressive symptoms became equivalent across the two groups. This model with all paths equal, all variances equal, and the independent variables equal showed good model fit X^2^(44) = 68.97, *p* < 0.01, RMSEA = 0.07, 90% CI (0.04, 0.09), CFI = 0.97, TLI = 0.97, and SRMR = 0.10. Those in older age group had lower social support (*M* = −0.47, *z* = 2.43, *p* = 0.02). In summary, the measurement invariance analysis showed that a model constraining the two groups to be equal, with the exception of the latent variable of social support, had the best model fit.

### Structural Equation Model for Health

**FIGURE 2:**
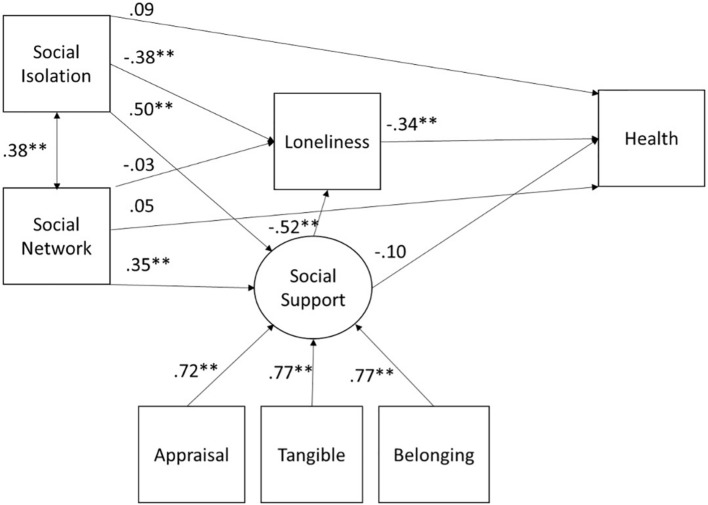
SEM for health for the full sample. All paths are denoted with the standardized beta. *p < 0.05, **p < 0.01.

The model tested for self-ratings of health was the same as for depressive symptoms (Figure 2). The model fits were generally excellent: X^2^(8) = 11.46, *p* = 0.18, RMSEA = 0.04 90% CI (0.00, 0.08), CFI = 1.00, TLI = 0.99, and SRMR = 0.01 (Table 4). The interrelationships of social support, social isolation, social network size, and loneliness were identical to those found for depression. Health was predicted only by loneliness with greater loneliness leading to worse self-ratings of health.

**TABLE 4:** Results of structural equation models for health.

Model	df	X^2^	SRMR	RMSEA	TLI	CFI	Δdf	ΔX^2^
**No age model**	8	11.46	0.014	0.038	0.99	0.998		
**Multi-group factor analysis**								
Model 1. Paths, means (LV, IV, DV), variances free	20	33.38*	0.043	0.067	0.97	0.98	–	–
Model 1 vs. Model 2	–	–	–	–	–	–	1	7.78**
Model 1 vs. Model 3	–	–	–	–	–	–	3	7.04
Model 2. Paths, means (IVs, DV), variances free	21	41.16**	0.082	0.080	0.95	0.97	–	–
Model 3. Paths, means (LV, DV), variances free	23	40.42*	0.047	0.071	0.96	0.98	–	–
Model 3 vs. Model 4	–	–	–	–	–	–	1	0.58
Model 3 vs. Model 5	–	–	–	–	–	–	1	8.21**
Model 4. Paths, mean LV, variances free	24	41.00*	0.047	0.069	0.96	0.98	–	–
Model 4 vs. Model 6	–	–	–	–	–	–	1	8.21**
Model 4 vs. Model 7	–	–	–	–	–	–	10	9.46
Model 5. Paths, mean DV, variances free	24	48.63**	0.090	0.083	0.95	0.97	–	–
Model 5 vs. Model 6	–	–	–	–	–	–	1	0.58
Model 6. Paths variances free	25	49.21**	0.091	0.080	0.95	0.97	–	–
Model 7. Variances, mean LV, free	34	50.46*	0.094	0.057	0.98	0.98	–	–
Model 7 vs. Model 8	–	–	–	–	–	–	8	13.40
Model *8. Mean LV Free*	*42*	63.86*	*0.10*	*0.059*	*0.97*	*0.97*	*–*	*–*

*The first model is without age. The rest of the models are multiple group analyses testing measurement equivalence on different sets of parameters. The best fitting multiple group model as determined by ΔX^2^ is in italic.*

*SRMR, Standardized Root Mean Square Residual; RMSEA, Root Mean Square Error of Approximation; TLI, Tucker-Lewis Index; CFI, Comparative Fit Index; Δdf, change in df; ΔX^2^, change in X^2^; LV, latent variable; IV, Independent Variable; DV, Dependent Variable.*

*p < 0.05, **p < 0.01.

Social isolation was not directly related to ratings of health (β = 0.09, *z* = 1.20, *p* = 0.23). However, there was a significant total indirect effect of social isolation (β = 0.17, *z* = −3.11, *p* = 0.002) on ratings of health through two paths: (1) through loneliness (β = 0.13, *z* = 2.79, *p* = 0.005), and (2) through social support and then through loneliness (β = 0.09, *z* = 2.36, *p* = 0.02). The indirect effect of social isolation to ratings of health through social support was not significant (β = −0.05, *z* = −0.70, *p* = 0.48) nor was the total indirect effect of social network size to social isolation (β = 0.04, *z* = 0.96, *p* = 0.34). However, the indirect effect of social network size to self-ratings of health was mediated through social support and then loneliness (β = 0.06, *z* = 2.13, *p* = 0.03). Finally, social support had a significant positive indirect effect on health through loneliness (β = 0.18, *z* = 2.55, *p* = 0.01).

In summary, those with higher levels of loneliness report worse health. In addition, the effect of social isolation on ratings of health was mediated by loneliness and social support. Further, social network size was significantly related to ratings of health via social support and loneliness.

### Multiple Groups Analysis of Health

We replicated the previous analysis with the two subgroups of older adults (aged 65–79 and 80–98). Table 4 shows model fit for all analyses, as with depression, the Table compares measurement equivalence with different levels of strictness criteria to establish both that the groups are comparable in terms of the structure of the model and what differences in levels exist. For ratings of health constraining the latent variable to be equal, significantly decreased model fit suggesting the two groups varied on the latent variable of social support. The model allowing the two groups to have different means for social support showed good fit X^2^(44) = 63.86, *p* = 0.02, RMSEA = 0.06, 90% CI (0.03, 0.09), CFI = 0.97, TLI = 0.97, and SRMR = 0.10. As observed for depression, the older group had lower scores on the latent social support variable (*M* = −0.46, *z* = 2.43, *p* = 0.02).

### Structural Equation Model for Cognition

**FIGURE 3:**
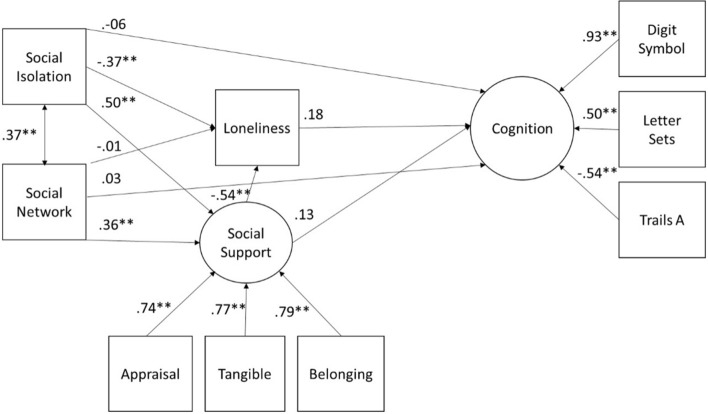
SEM for latent Cognition for the full sample. All paths are denoted with the standardized beta. *p < 0.05, **p < 0.01.

The model tested for cognition was the same as for depressive symptoms and health (Figure 3). Model fit was excellent [X^2^(20) = 25.91, *p* = 0.17, RMSEA = 0.03 90% CI (0.00, 0.06), CFI = 0.99, TLI = 0.99, and SRMR = 0.03]. The interrelationships of social support, social isolation, social network size, and loneliness were identical to those found for depression. Cognition was not significantly predicted by any of the social variables and there were no significant indirect effects.

### Multiple Groups Analysis of Cognition

We replicated the previous analysis with the two subgroups of older adults (aged 65–79 and 80–98). Table 5 shows model fit for all analyses conducted to establish to what degree the groups are equivalent and sources of differences should they exist. For ratings of cognition, constraining the latent variable of social support to be equal, significantly decreased model fit suggesting the two groups varied on the latent variable of social support; however, this was not the case for the latent variable of cognition. As observed for depressive symptoms and health, the older group had lower social support (*M* = −0.47, *z* = 2.59, *p* = 0.01). The best fitting model indicated the paths to depressive symptoms to be significantly different between the two groups. This model showed good fit [X^2^(69) = 93.37, *p* = 0.03, and RMSEA = 0.049 90% CI (0.02, 0.07), CFI = 0.97, TLI = 0.97 and SRMR = 0.14] (see Table 5 for complete results). Probing the paths to cognition showed that the paths from social network size and [X^2^(1) = 3.98, *p* = 0.03], and social isolation [X^2^(1) = 6.81, *p* = 0.01] were significantly different between the two age groups if tested separately or together [X^2^(2) = 10.94, *p* = 0.004]. However, the parameter estimates for the individual group paths for social isolation and cognition were not significant for the younger group, (*b* = 0.29, *z* = 1.02, *p* = 0.31 or the older, *b* = −0.49, *z* = −1.43, *p* = 0.15). For social network size the effect was significant for the older group (*b* = 0.41, *z* = 2.27, *p* = 0.02), but not significant for the younger group (*b* = 0.04, *z* = −0.63, *p* = 0.53). For the older group a having a larger social network was related to higher cognition.

**TABLE 5:** Results of structural equation models for cognition.

Model	df	X^2^	SRMR	RMSEA	TLI	CFI	Δdf	ΔX^2^
**No age model**	20	23.15	0.021	0.023	0.994	0.997		
**Multi-group factor analysis**								
Model 1. Paths, means (LV, IV, DV), variances free	48	61.33	0.051	0.043	0.98	0.99	–	–
Model 1 vs. Model 2	–	–	–	–	–	–	1	7.70**
Model 1 vs. Model 3	–	–	–	–	–	–	3	7.00
Model 2. Paths, means (IVs, DV), variances free	49	69.03*	0.076	0.052	0.97	0.98	–	–
Model 3. Paths, means (LV, DV), variances free	51	68.33	0.055	0.048	0.98	0.98	–	–
Model 3 vs. Model 4	–	–	–	–	–	–	1	0.14
Model 3 vs. Model 5	–	–	–	–	–	–	1	8.15**
Model 4. Paths, mean LV, variances free	52	68.47	0.055	0.046	0.98	0.98	–	–
Model 4 vs. Model 6	–	–	–	–	–	–	1	8.06**
Model 4 vs. Model 7	–	–	–	–	–	–	10	19.21**
Model 4 vs. Model 8	–	–	–	–	–	–	11	16.45
Model 5. Paths, mean DV, variances free	52	76.48*	0.083	0.056	0.97	0.97	–	–
Model 5 vs. Model 6	–	–	–	–	–	–	1	0.05
Model 6. Paths, variances free	53	76.53*	0.083	0.054	0.97	0.98	–	–
Model 7. Variances, mean LV, free	62	87.68*	0.091	0.053	0.97	0.97	–	–
Model 8. Paths, mean LV free	63	84.92*	0.133	0.048	0.97	0.98	–	–
Model 8 vs. Model 9	–	–	–	–	–	–	10	19.60**
Model 8 vs. Model 10	–	–	–	–	–	–	6	8.44
Model 9. Mean LV free	73	104.52**	0.127	0.054	0.97	0.97	–	–
Model 10. Cognitive paths, mean LV free	69	93.362*	0.14	0.05	0.97	0.97	–	–

*The first model is without age. The rest of the models are multiple group analyses testing measurement equivalence on different sets of parameters. The best fitting multiple group model as determined by ΔX^2^ is in italic.*

*SRMR, Standardized Root Mean Square Residual; RMSEA, Root Mean Square Error of Approximation; TLI, Tucker-Lewis Index; CFI, Comparative Fit Index; Δdf, change in df; ΔX^2^, change in X^2^; LV, latent variable; IV, Independent Variable; DV, Dependent Variable in this case latent cognition.*

*p < 0.05, **p < 0.01.

### Multiple Regressions Predicting Loneliness and Social Isolation

As loneliness and social isolation were pivotal variables in our model, we decided to more thoroughly test the factors that predicted both constructs. Guided by the WHO’s international Classification of Function, Disability, and Health (ICF; World Health Organization, 2002) model, we included three sets of potential predictors, personal factors (age, gender, income, education, and race), environmental factors (life space, social network size, and life engagement), and health (reported number of functional limitations, and number of reported health conditions) on the sample of 253 participants for whom we had complete data. We eliminated 13 participants as they identified their race/ethnicity as other than White, Hispanic, or African American (e.g., Asian, Mixed Race) and there were too few participants in the other categories for meaningful comparisons. To confirm that each of these sets were important we ran sequential hierarchical multiple regressions (Table 6).

**TABLE 6:** Descriptive statistics for hierarchical variables included in the multiple regression analyses (*N* = 253).

Variable	Statistic
	M (SD)
Loneliness	39.3 (9.7)
Friendship	19.3 (7.4)
Age	75.7 (7.4)
Functional limitations	5.6 (3.2)
Health conditions	3.3 (1.7)
Life space	5.7 (1.5)
Social network size	26.4 (7.3)
Life engagement	24.9 (4.0)
Functional limitations	5.6 (3.2)
Health conditions	3.3 (1.7)
	n (%)
Gender	
Female	196 (76.7%)
Male	59 (23.3%)
Income	
<$30000	218 (86.2%)
30000-$59999	31 (12.2%)
>$59999	4 (1.6%)
Race	
African-American	92 (36.4%)
Hispanic	26 (10.3%)
White	135 (53.3%)

The full model for loneliness was strongly significant *F*(10,242) = 20.23, *p* < 0.001, *R*^2^ = 0.46. The step entering personal factors explained the least variance and was not statistically significant [*F*(5,247) = 1.33, *p* = 0.25, *R*^2^ = 0.03], and thus we do not discuss individual parameters (see Table 7). In the second step, we entered the health variables and this step was strongly statistically significant [*F*(2,245) = 19.18, *p* < 0.001, Δ*R*^2^ = 0.13]. The only significant variable was functional limitations; having more limitations was associated with higher degrees of loneliness [*t*(245) = 4.23, *p* < 0.001, *f*^2^ = 0.07]. In the third step, we entered the environmental variables and this step also explained a great deal of variance *F*(3,242) = 44.02, *p* < 0.001, Δ*R*^2^ = 0.30. Having a larger social network was associated with less loneliness [*t*(242) = −4.85, *p* < 0.001, *f*^2^ = 0.10], and higher reporting of engaging in valued activities was also associated with less loneliness [*t*(242) = −8.34, *p* < 0.001, *f*^2^ = 0.29].

**TABLE 7:**
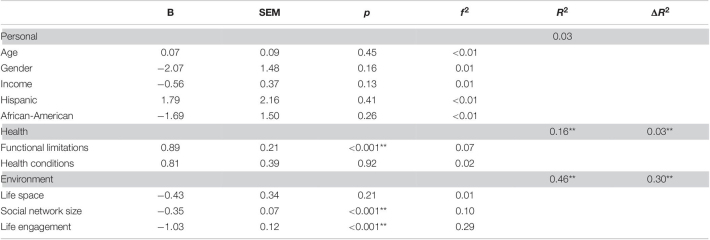
Results of hierarchical regression predicting loneliness.

*The row in gray represents a set of variables with R^2^ being the variance explained by all variables in the model at the step and ΔR^2^ being the change in variance explained by the set of variables added in the step. Parameter estimates are for the step.*

*B, Unstandardized Beta; SEM, Standard error of measurement; f^2^, Cohen’s f^2^ a measure of effect size representing the signal to noise ratio.*

*p < 0.05; **p < .01.

The full model for social isolation was also strongly significant [*F*(10,242) = 12.53, *p* < 0.001, *R*^2^ = 0.34]. The step entering personal factors explained the least variance and was not statistically significant [*F*(5,247) = 1.42, *p* = 0.22, *R*^2^ = 0.03], thus we do not discuss individual parameters (see Table 8). In the second step, we entered the health variables and this step was strongly significant [*F*(2,245) = 8.91, *p* < 0.001, Δ*R*^2^ = 0.07]. Having more functional limitations with more limitations was associated with greater social isolation [*t*(245) = −2.76, *p* < 0.001, *f*^2^ = 0.03]. In the third step, we entered the environmental variables and this step also explained a great deal of variance [*F*(3,242) = 30.25, *p* < 0.001, Δ*R*^2^ = 0.25] in social isolation. Again, having a large social network was associated with less loneliness [*t*(242) = 3.56, *p* < 0.001, *f*^2^ = 0.05], as did higher reporting of engaging in valued activities [*t*(242) = 7.05, *p* < 0.001, *f*^2^ = 0.21].

**TABLE 8:**
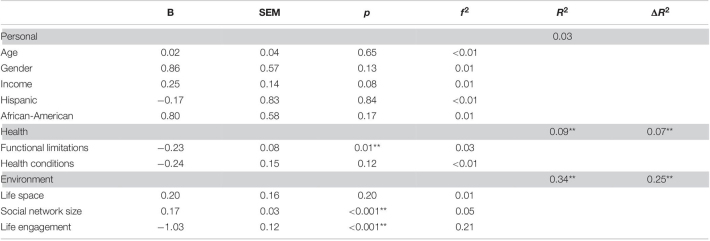
Results of hierarchical regression predicting social isolation.

*The row in gray represents a set of variables with R^2^ being the variance explained by all variables in the model at the step and ΔR^2^ being the change in variance explained by the set of variables added in the step.*

*B, Unstandardized Beta; SEM, Standard error of measurement; f^2^, Cohen’s f^2^ a measure of effect size representing the signal to noise ratio.*

*p < 0.05, **p < 0.01.

## Discussion

The aging of the population generates a pressing need to develop strategies to ensure that current and future cohorts of older people are able to live as independently as possible and enjoy a good quality of life. Recently, increased attention is being directed toward social isolation and loneliness among older people, as being isolated and lonely has a deleterious impact on physical, emotional, and cognitive health. Current data indicate that a large proportion of the aging population is socially isolated and lonely (e.g., Anderson and Thayer, 2018; Cudjoe et al., 2020). Strategies to prevent or remediate social isolation and loneliness are predicated on understanding factors that are related to being socially isolation and lonely. This is a complex issue as social engagement has many components, which are correlated but distinct.

In this study we had the unique opportunity to examine the relationships among a number of constructs related to loneliness and social isolation among a diverse sample of older adults living alone in the community. We explored the relationships between loneliness and isolation on depressive symptoms, health, and cognitive outcomes. Our sample included both younger-old and older-old individuals thus we could explore if these relationships varied according to these subgroups of older people. Our findings help to clarify the relationships among the various aspects of social support, isolation, and loneliness, as well as the resultant impacts on both mental health, physical health, and cognition. Further, we evaluated personal, environmental, and health factors that are associated with isolation and loneliness.

Consistent with models of successful aging (e.g., Rowe and Kahn, 1988; Kahana and Kahana, 1996, 2001), our findings indicated that social engagement is an important aspect of what it means to age successfully. Overall, the results underscored the findings of other investigators (e.g., Cacioppo et al., 2010; Aylaz et al., 2012; Valtorta et al., 2016; Hakulinen et al., 2018; Jeuring et al., 2018; Domenech-Abella et al., 2019; Read et al., 2020), that loneliness and social isolation have a significant impact on emotional well-being and health. Loneliness is particularly deleterious as it has a direct impact on both emotional and physical health. People who were lonely were more likely to report depressive symptoms and rated their health as worse than those who were not lonely. This has important implications for older adults and society as a whole. The economic burden of depression in the United States is about $210 billion annually, which includes costs associated with the treatment of depression itself as well as associated co-morbidities. In addition, those who reported that they had limitations engaging in meaningful activities and those with more functional limitations reported greater loneliness.

Our results showed that that loneliness was significantly related to symptoms of depression. There was also a significant direct path between social isolation and depressive symptoms. These findings suggest that isolation and loneliness are related but distinct constructs. Not surprisingly, social isolation was predicted by social network size. Social network size and social isolation were also related to social support, which in turn was related to loneliness. In general, older adults with larger social networks were less likely to be isolated and had greater perceived social support. They were also less likely to be lonely. Our findings showed that among the older adults, social network size was also related to cognition such that people in the older cohort with larger social networks scored higher on the composite measure of cognition. As noted, having a larger social network likely provided more opportunities for engagement and support. Alternatively, maintaining a social network may require a certain amount of cognition and individuals with higher levels of cognitive function may be better able to maintain those relationships. Others have found that size of one’s social network is also related to access to resources (e.g., Cannuscio et al., 2003). The majority of individuals in our sample were in the lower socio-economic strata.

With respect to self-ratings of health, we found slightly different relationships. Specifically, loneliness had a direct negative impact on health but the relationship between social isolation and ratings of health was mediated by loneliness and social support. Although our study was limited to a subjective rating of health, others (i.e., Wu et al., 2013) have found that self-ratings of health are predictive of objective indices of health and mortality. Thus, our findings underscore the importance of social engagement to health and well-being.

We did not find differences in the relationships among the variables for depressive symptoms or ratings of health between the younger-old and older-old adults. However, we did find that perceived social support was lower among the older-old people in our sample. This is important given the increasing number of people in this cohort and the relationship between social support and loneliness. People in the older age cohorts, especially older women are more likely to live alone and have fewer sources of support available due to changes in life circumstances.

The findings from our regression analyses also point to the associations among individual and environmental variables and social isolation and loneliness. We found, not surprisingly that individuals with more functional limitations and those who less engaged in rewarding activities reported higher levels of loneliness. It is likely that functional limitations result in logistic hindrances to activity engagement.

Our findings have important implications for the design of interventions. Strategies to increase the social networks older adults, enhance social support, and the ability to engage in meaningful and enjoying activities would likely be beneficial in terms of improving health outcomes, especially for those with functional limitations. These interventions might include creating affordable programs for older adults and connecting individuals to these programs or venues for peer support. It might also involve facilitating access to transportation services and enhancing community safety.

Information and communication technologies offer vast potential in terms of promoting social engagement (Bixter et al., 2018). For example, social media platforms such as Facebook and LinkedIn, offer opportunities to make new friends and share information about life events with friends and family thus promoting connectivity and a sense of belonging. Data from the Pew Research Center (Anderson and Perrin, 2017) indicate that older adults are increasingly using social media platforms to share their experiences and connect with friends and family (see also Bixter et al., 2019). Video chat platforms provide an additional avenue for social communication and cognitive enrichment (e.g., Nie et al., 2020) as well as physical activity (Beer et al., 2015).

Access to the Internet and email can also foster social connectivity. Findings from the PRISM trial (Czaja et al., 2018) found that use of the PRISM software system resulted in decreased loneliness among older people. One of the most used PRISM features was the internet and one of the reported benefits of PRISMs was the ability to communicate with families and friends. Others have also found that having access to the Internet benefits social engagement (e.g., Cotten et al., 2013; Morris et al., 2014). A recent study (Wu, 2020) found that internet use was associated with decreased loneliness over an 8-year period as it was a vehicle for maintaining social contact.

Virtual reality (VR) applications are increasingly being targeted toward older adults and provide a mechanism for interacting with others in an individual or group format as well as engaging in valuable activities. For example, one can engage in virtual travel or cultural events using a VR system. As noted, our data indicate that lack of engagement in valued activities is related to both social isolation and loneliness. These applications may also be especially beneficial for those with functional limitations who may have mobility restrictions. Design recommendations for VR systems targeted to older adults are being developed (e.g., McGlynn and Rogers, 2017). Developments in robotics are being geared toward enhancing social interactions among older adults (Rogers and Mitzner, 2017).

However, although technology holds great potential in terms of fostering social interactions and decreasing loneliness among older adults it is important that technology applications are designed using a user-centered design approach where diverse and representative samples of older adult users are involved in the design process. This approach helps to ensure that the needs, preferences, and characteristics of aging adults are incorporated into the design of the technology application. Aging adults must be aware of technology developments and how various technology applications may improve their well-being and quality of life. Technology must be affordable and instructional and technical support must be available. Finally, technology cannot replace human contact, it provides a complementary vehicle for social interaction.

Importantly, simply increasing social networks and social engagement is not sufficient for decreasing loneliness. The networks and engagement must be satisfying and result in enhanced feelings of support. Activities must also be rewarding and engaging. Finally, it is important to recognize that there is no one-size-fits-all approach to addressing loneliness or social isolation, and tailor interventions should be tailored to the needs, preferences, and contexts of individuals.

Limitations of this study include the use of a self-report single item measure of health. Although this measure is commonly used and has been found to be related to objective health metrics. In addition, the data were cross-sectional and from a single time point which reduces the ability to make causal inferences. Our sample was largely of lower socio-economic status and restricted to individuals who lived alone in the community. Further, our sample was a convenience sample, that agreed to participate in a research trial. Thus, the findings may not generalize to other subpopulations of older adults. Finally, although path models are useful in conceptualizing interrelationships among variables of interest, these models only present associations and do not prove causal relationships. Despite these limitations the present study adds to the growing body of literature examining the important role of social engagement in promoting health and well-being among older people. It clearly demonstrated associations and pathways among social isolation, social support, and loneliness. We recognize of course that these relationships may be bi-directional or in the opposite direction hypothesize, for example that social isolation may be affected by health, which underscores the complexity of these relationships. Nonetheless, these data provide valuable guidance for the development of interventions to both prevent isolation and loneliness among those who are at risk and remediate these problems for those who are currently isolated and lonely. The findings also underscore the importance of directing attention to the public health risk of social isolation and loneliness especially in light of the COVID-19 pandemic.

## Data Availability Statement

The raw data supporting the conclusions of this article will be made available by the authors, without undue reservation.

## Ethics Statement

The studies involving human participants were reviewed and approved by the Miller School of Medicine IRB. The patients/participants provided their written informed consent to participate in this study.

## Author Contributions

SC and WR designed the study that is the source of the article. SC drafted the manuscript. JM designed the analysis, conducted and written in consultation with SC. All authors contributed to editing the article.

## Conflict of Interest

The authors declare that the research was conducted in the absence of any commercial or financial relationships that could be construed as a potential conflict of interest.

## Publisher’s Note

All claims expressed in this article are solely those of the authors and do not necessarily represent those of their affiliated organizations, or those of the publisher, the editors and the reviewers. Any product that may be evaluated in this article, or claim that may be made by its manufacturer, is not guaranteed or endorsed by the publisher.
